# Design and Development of Sectional-Chain Silicon Drift Detectors with Diversity Elliptical-Shaped Voltage Dividers

**DOI:** 10.3390/mi17050549

**Published:** 2026-04-29

**Authors:** Chunxiang Ni, Tao Long, Jun Zhao, Xuyang Song, Xinqing Li, Manwen Liu, Zhiyu Liu, Xuran Zhu, Zheng Li

**Affiliations:** 1 School of Integrated Circuits, Ludong University, Yantai 264025, China; 13465506066@163.com (C.N.); zhaojun@ldu.edu.cn (J.Z.); 16634270575@163.com (X.S.); 17861822572@163.com (Z.L.); 18663488310@163.com (X.Z.); 2 Engineering Research Center of Photodetector Special Chip in Universities of Shandong, Ludong University, Yantai 264025, China; xqli1996zz@163.com; 3 School of Materials Science and Engineering, Xiangtan University, Xiangtan 411105, China; 4 Institute of Microelectronics, Chinese Academy of Sciences, Beijing 100029, China; liumanwen@ime.ac.cn

**Keywords:** silicon drift detector, resistive voltage divider, sectional chain, electrical performance

## Abstract

This paper proposes a sectional-chain silicon drift detector (SDD), featuring an elliptical-shaped voltage divider resistor chain, that can address the issue with traditional concentric ring SDDs, which cannot independently provide voltage division. The study replaces the conventional linear voltage divider with an elliptical structure, using its diversity geometry to improve the uniformity of the electric field distribution within the detector’s sensitive area, effectively solving the problem of distortions of edge electric fields and those between the SDD’s cathode rings in traditional structures. The relevant parameters of the elliptical resistor chain are calculated through formulas, which establish a quantitative relationship between the resistance values and the elliptical geometric dimensions, providing a theoretical basis for electric field uniformity control. A device physics model is then established using TCAD for simulation analysis to obtain key performance parameters: the electric potential and electric field distribution inside the detector, the spatial distribution of electron concentration in the detector bulk, and the electric potential gradient on the detector surface. These parameters provide a design reference for the application of high-performance SDDs in fields such as X-ray energy spectroscopy nuclear physics experiments and space radiation monitoring.

## 1. Introduction

Silicon drift detectors (SDDs) are detectors capable of detecting X-rays and charged particles with high energy resolutions [1,2]. With their unique structure, excellent performance, and mature fabrication process [3,4], they are widely used in fields such as material sciences, synchrotron radiation, space and astronomical physics [5]. The core principle of an SDD is to use a lateral electric field to direct the electrons (or holes) generated by ionizing radiation to drift toward a small central collection anode [6,7], achieving both high energy resolution and high count rates [7,8,9,10,11].

**Figure 1:**
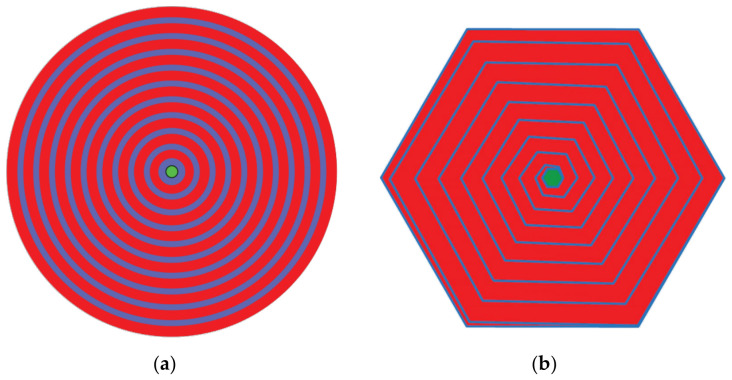
(**a**) Concentric ring electrode SDD; (**b**) spiral electrode SDD.

Traditional circular-type SDDs are divided into concentric ring and spiral ring types [12], each with its drawbacks. The traditional concentric ring electrode SDD requires applying a given voltage to each ring to establish a lateral potential gradient (as shown in Figure 1a), which is usually implemented by an external resistor chain [13,14]. However, due to the need for extremely high resistive matching accuracy, this external resistor chain is hard to come by and even more difficult to interconnect to the SDD’s rings. On the other hand, spiral ring electrode SDDs (as shown in Figure 1b), although using p+-rings in the spiral cathode rings as both resistor chain to provide self-bias for voltage division and rectifying junctions, it results in a more complex structural design [15,16,17]. Furthermore, due to the reduced structural symmetry of spiral electrode SDDs, their potential distribution may not be as uniform as that of concentric ring electrode SDDs [18,19,20].

In this work, we propose a novel sectional-chain silicon drift detector (SDD) design based on a concentric ring SDD with a diversity elliptical voltage divider resistor chain. This detector can achieve self-voltage division without relying on an external voltage dividing chain, addressing the shortcomings of traditional concentric ring SDDs [21,22]. In this study, the elliptical voltage divider resistor chain between the cathode rings is calculated using a mathematical iterative formula. The core principle is to ensure that the resistance between adjacent cathode rings is the same and the resistor chain occupies the entire gap area between SDD rings, resulting in a more uniform voltage gradient distribution and more uniform electric field profiles. The resistor chain is connected to the cathode rings through P-type doping, allowing the detector to achieve self-voltage division. Meanwhile, the total number of the elliptical arc segments for the nth ring, *m_N_*, and the width of the elliptical arc w_n_ determine the resistance between the rings in the detector. This elliptical voltage divider design provides better uniformity of the drift electric field, which is expected to significantly improve charge collection efficiency and energy resolution, and may simplify the design of the signal readout electronics board.

## 2. Detector and Biasing Resistor Chain Design and Calculation

**Figure 2:**
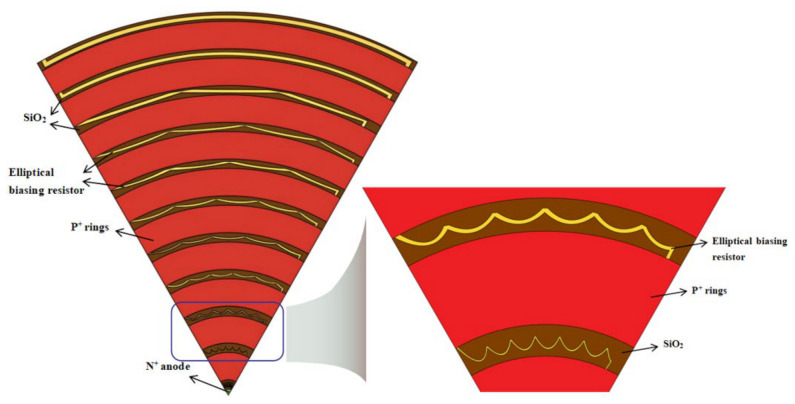
Design schematic of a 1/6 circle (θ = π/3).

In the calculation process designed here, we use a sector (1/6 of a circle that is a section of our sectional-chain SDD) for the computation. The top view of this design is shown in Figure 2. When changing the sectional angle θ (in this case θ = π/3), the corresponding results will change automatically. In this way, different shapes of detectors with different section angles θ and total numbers of sections (k, with kθ ≤ 2π) can be assembled. Therefore, the calculation method in this design can be regarded as suitable for a single sector (unit sector) of a whole SDD. Using this approach, detectors suitable for different scenarios can be obtained.

In the experiment, a sectional-chain silicon drift detector with elliptical resistive voltage dividers is designed and fabricated using a 300 μm thick n-type ultra-pure high-resistance silicon wafer. Essentially, the designed sector can be assembled maximally into a cylinder with an SDD diameter of 5 mm and a height of 300 μm. The center of the top surface is n^+^-doped to serve as the collecting anode. The anode has a radius of 60 μm and a depth of 0.5 μm. The ion implantation (boron, p^+^) depth for the elliptical resistor chain between the cathode rings is 0.5 μm, and the cathode is p^+^-doped with an ion implantation depth of 1 μm. The resistors are connected in a linear fashion, and the spacing between rings and the width of the resistor chain between rings are determined through iterative calculations. This design almost fully occupies the gap between the SDD rings, effectively reducing the depleted area between rings, and therefore reducing the detector’s surface leakage current, thereby lowering the detector noise. More importantly, it improves upon the drawbacks of traditional concentric ring voltage boosting. Figure 2 shows a schematic of the 1/6 circle design, with an 80 μm gap reserved between the innermost cathode ring and the central collecting anode, ensuring the detector can be conveniently connected to readout electronics. The back surface of the detector is covered with a 1 μm deep boron (p^+^) ion implantation, providing a uniform radiation entrance window. A bias voltage VB is applied to the back of the detector; a bias voltage V_out_ is applied to the outermost ring of the detector; and a bias voltage V_E1_ is applied to the first cathode ring. These voltages control the internal electron drift channel of the detector.

**Figure 3:**
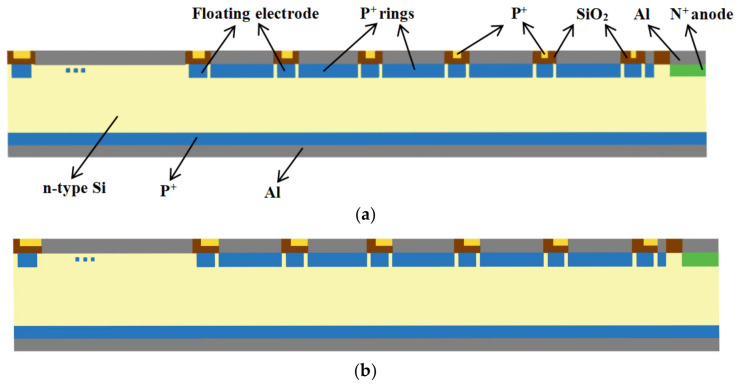
(**a**) Cross-sectional view of the detector; (**b**) cross-sectional view of the connection between the detector’s resistor chain and the cathode ring.

Figure 3a shows a cross-sectional view of the detector, where it can be seen that the resistive chain is located between the two cathode rings. Figure 3b displays a cross-sectional view at the connection between the resistive chain and the cathode ring, where the resistive chain connects to the aluminum (1 μm thick) layer above the cathode ring, facilitating conductivity and improving voltage division. The thickness of the SiO_2_ layer is 1 μm.

**Figure 4:**
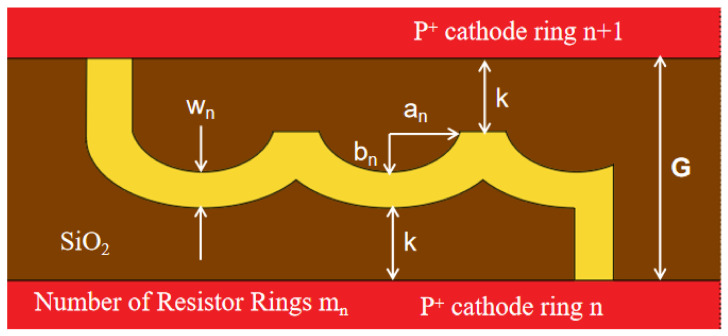
Reference chart of formula letters.

The derivation method of the elliptical-shaped resistor chain parameters for the sectional-chain silicon drift detector designed in this paper is initiated and optimized in this work. Taking a 1/6 circle as an example, the derivation formula for each resistive ring is as follows. The definitions of notations in the formula are given and shown in Figure 4. The width of the resistor chain in the innermost ring, *w*_1_, is set to 5 μm, which can be reduced according to the processing capability and application needs.

The width of the gap between two neighboring cathode rings G, where the resistor chain is located, is set at a constant 60 μm for all gaps. The distance between the edge of the elliptical resistor arc and that of the resistor chain of the adjacent two cathode rings, *k*, is set at a constant 10 μm. The ratio ε of the innermost resistor chain width *w*_1_ to the outermost resistor chain width *w_N_* is set at 0.2. The maximum radius of the detector cathode ring, *r_N_*, is set at 2500 μm and the associated number of elliptical arcs is set at *m_N_* = 2. The radius of the innermost ring, *r*_1_,where the resistor chain starts, is set at 100 μm.

From all these settings, the minor axis of a single arc (half of an ellipse) in the first elliptical resistor chain (*b*_1_) and the width of the outermost elliptical resistor chain (*w_N_*) can be determined:

(1) $$b_{1}\;=\;G\;-\;2k\;-\;\frac{1}{2}w_{1}=\;37.5\;\mu m$$

(2) $$w_{N}=\;\frac{w_{1}}{\varepsilon }=25\;\mu m$$

The minor and major axes of a single arc in the outermost elliptical resistor chain are

(3) $${\;b}_{N}=G-2k-\frac{1}{2}w_{N}=27.5\mu m$$

(4) $$a_{N}=\frac{r_{N}\theta }{2m_{N}}=\frac{2500\mu m\cdot \frac{\pi }{3}}{2\cdot 2}=654.5\mu m$$

when calculating the length of an elliptical resistor chain, the formula used for calculating the circumference of the ellipse is

(5) $$l_{n}=\pi \sqrt{2\left(a_{n}^{2}+b_{n}^{2}\right)}\;(n=1,2,\ldots \;N)$$

Therefore, by substituting the results calculated in (3) and (4) into (5), the circumference of the outermost ellipse can be obtained:

(6) $$l_{n}=\pi \sqrt{2\left(a_{n}^{2}+b_{n}^{2}\right)}=2912.64\mu m$$

Since it is necessary to ensure that the resistance of every elliptical resistor chain between the cathode rings is equal to the others to achieve uniform voltage drop (or gradient) between cathode rings, it can be concluded that the elliptical resistor chain resistance (*R*_1_) between the first and second rings (*r*_1_ and *r*_2_) is

(7) $$R_{1}=\frac{m_{1}l_{1}}{w_{1}}=\frac{m_{N}{\;l}_{N}}{w_{N}}=R_{N}$$

and its total length *l*_1_ is

(8) $$l_{1}=\frac{m_{N\;}l_{N}{\;w}_{1}}{m_{1}w_{N}}=\frac{m_{N}l_{N}\varepsilon }{m_{1}}$$

From the derivation of (7), it can be obtained that

(9) $$\frac{m_{1}}{m_{N}}=\frac{w_{1}{\;l}_{N}}{w_{N}\;l_{1}}=\varepsilon \frac{l_{N}}{l_{1}}$$

Also, because the calculation formula for the number of elliptical arcs *m*_1_ of the first-stage elliptical resistor chain is

(10) $$m_{1}\;≅\frac{r_{1}\theta }{2a_{1}}$$

From (8)–(10), it can be seen that the expression for *l*_1_ is

(11) $$l_{1}=\frac{2\varepsilon m_{N}{\;l}_{N}}{r_{1}\theta }a_{1}=\alpha a_{1}\;(\alpha =\frac{2\varepsilon m_{N}l_{N}}{r_{1}\theta }=22.25)$$

Also, because the formula for the circumference of an ellipse is

(12) $$l_{1}=\pi \sqrt{2\left(a_{1}^{2}+b_{1}^{2}\right)}$$

From (10) and (11), the short axis *a*_1_ of the innermost resistor chain can be obtained:

(13) $$a_{1}=\;\frac{l_{1}}{\alpha }\;=\;\frac{\pi \sqrt{2\left(a_{1}^{2}+b_{1}^{2}\right)}}{\alpha }$$

We have

(14) $$a_{1}=\sqrt{\frac{2}{{\left(\frac{\alpha }{\pi }\right)}^{2}\;-\;2}}\;b_{1}=7.65\mu m$$

The number of arcs in the first loop of the resistor chain is

(15) $$m_{1}=\frac{r_{1}\theta }{2a_{1}}=7$$

and

(16) $$l_{1}=\alpha a_{1}=170.2\;\mu m$$

At this point, all the parameters of the innermost resistor chain have been completely obtained (*a*_1_ = 7.65 μm, *b*_1_ = 37.5 μm, *l*_1_ = 170.2 μm, *m*_1_ = 7, and *w*_1_ = 5 μm). Next, the data for the subsequent resistor chains is calculated using the iterative method. In this paper, the calculation is carried out by reducing the number of arcs per resistor chain by half for each subsequent circle; that is, m is 0.5. The first iterative calculation of the second stage of resistors is as follows:

(17) $$m_{2}=m_{1}+1$$

(18) $$w_{2}^{\left(1\right)}=\frac{m_{1}+1}{m_{1}}w_{1}=5.714\;\mu m$$

(19) $$b_{2}^{\left(1\right)}=G-2k-\frac{1}{2}w_{2}^{\left(1\right)}=37.143\;\mu m$$

(20) $$a_{2}=\frac{r_{2}\theta }{2\cdot m_{2}}=50.615\;\mu m$$

(21) $$l_{2}^{\left(1\right)}=\pi \sqrt{2\left(a_{2}^{2}+b_{2}^{\left(1\right)}\right)}=278.93\;\mu m$$

(22) $$\varepsilon_{2}^{\left(1\right)}=\frac{m_{1}l_{1}}{m_{2}l_{2}^{\left(1\right)}}=0.7112$$

The second iteration calculation is

(23) $$w_{2}^{\left(2\right)}=\frac{w_{1}}{\varepsilon_{2}^{\left(1\right)}}=7.030\;\mu m$$

(24) $$b_{2}^{\left(2\right)}=G\;-\;2k\;-\;\frac{1}{2}w_{2}^{\left(2\right)}=36.485\;\mu m$$

(25) $$l_{2}^{\left(2\right)}=\pi \sqrt{2\left(a_{2}^{2}+{\;b}_{2}^{\left(2\right)2}\right)}=277.2\;\mu m$$

(26) $$\varepsilon_{2}^{\left(2\right)}=\frac{m_{1}l_{1}}{m_{2}l_{2}^{\left(2\right)}}=0.7123$$

The third iteration calculation is

(27) $$w_{2}^{\left(3\right)}=\frac{w_{1}}{\varepsilon_{2}^{\left(2\right)}}=7.019\;\mu m$$

(28) $$b_{2}^{\left(3\right)}=G-2k-\frac{1}{2}w_{2}^{\left(3\right)\;}=36.491\;\mu m$$

(29) $$l_{2}^{\left(3\right)}=\pi \sqrt{2\left(a_{2}^{2}+b_{2}^{\left(3\right)2}\right)}=277.23\;\mu m$$

(30) $$\varepsilon_{2}^{\left(3\right)}=\frac{m_{1}l_{1}}{m_{2}l_{2}^{\left(3\right)}}=0.7162$$

When the iteration reaches an error limit of less than 0.1%, it is considered that the preset accuracy value has been reached and the iteration stops. The iteration method for subsequent stages of elliptical resistor chains are the same as the above process and will not be elaborated here further.

The detector’s full depletion voltage can be calculated using

(31) $$V_{fd}=\frac{qN_{eff}d^{2}}{2\varepsilon_{o}\varepsilon_{si}}$$

where *q* is the electronic charge, *q* = 1.6 × 10^−19^ C; *N_eff_* is the effective doping concentration of the silicon substrate (*N_eff_* = 1 × 10^12^ cm^−3^); d is the thickness of the silicon substrate (*d* = 300 µm); *ε*_0_ is the vacuum permittivity (*ε*_0_ = 8.854 × 10^−12^ F/m); and *ε_si_* is the relative permittivity of silicon (*ε_si_* = 11.9). The full depletion voltage in the detector for this work is approximately 68.3 V.

## 3. Full 3D Detector Modeling and Simulation

**Figure 5:**
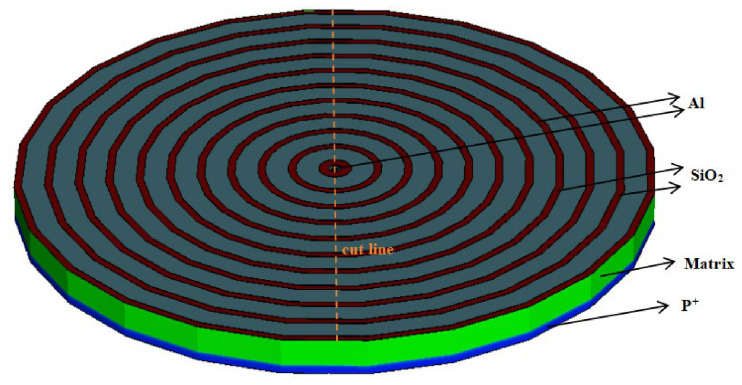
3D model of a full silicon drift detector (θ = 2π) with elliptical resistor biasing chains.

A model of a completed detector (θ = 2π) was created using the Sentaurus TCAD2019 semiconductor simulation software for the purpose of facilitating the simulation procedure (less boundary conditions applied). Taking the 3D model of a 10-ring silicon drift detector as an example, as shown in Figure 5, the gray areas on the surface of the detector are aluminum electrode contact layers. Only the aluminum at the very center and the outermost ring require applied voltages, while the aluminum layers in all other rings are used solely for conductivity. Neighboring cathode P^+^ rings are connected by respective elliptical resistor chains (as designed in the previous section) between them. Additionally, a cutting line is defined as shown in the figure to facilitate subsequent cross-sectional analysis.

**Figure 6:**
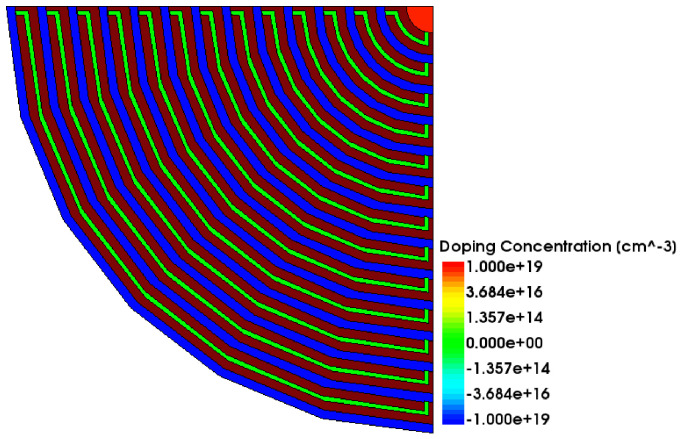
Surface model of a full silicon drift detector (θ = 1/2π).

In order to better display the details of the detector surface, one quarter of the detector is cut out, as shown in Figure 6. The top of the detector shows different colors due to different doping concentrations. The red ring at the center of the detector is the n-type collecting anode with a phosphorus doping concentration of 10^19^/cm^3^, the blue ring is the p-type cathode ring with a boron doping concentration of 10^19^/cm^3^, and the green is the resistor chain used for voltage division.

**Figure 7:**
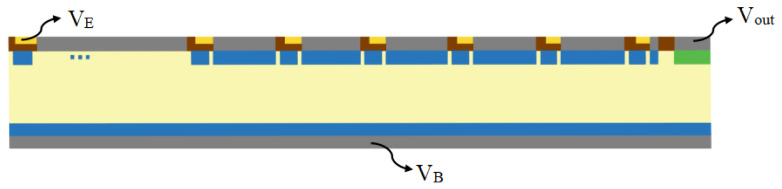
Specific methods of applying voltage in electrical characteristic simulations.

The parameters adopted in this study are based on experimental experience and are expected to yield favorable experimental results. In the study of electrical characteristics, it is necessary to apply pressure to the detector. The novel silicon drift detector introduced in this study requires pressure to be applied at only three locations, which significantly reduces the difficulty of operation. The specific pressure application method is shown in Figure 7.

Among the 10-ring detectors studied in this paper, the size of the central collection anode is already small relative to the detector’s surface area. This ensures sufficiently low capacitance, and the area of the collection anode can also be adjusted by optimizing the distance between the innermost cathode ring and the central collection anode. Such adjustments should make sure that the parameters do not cause detector breakdown or short-circuiting. The parameters used in this study are based on experiment experience and can achieve good experimental results.

### 3.1. Detector Electric Potential Distribution

**Figure 8:**
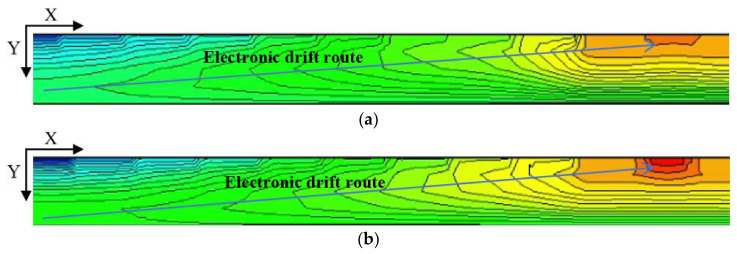
(**a**) Potential profile at one times the depletion voltage; (**b**) potential profile at two times the depletion voltage.

Using the cutting line shown in Figure 5, we can obtain the 2D electric potential distribution map from 3D-simulation data, as shown in Figure 8. The SDD bias conditions are as the follows: (a) the anode is biased at 0 V, (b) the innermost cathode ring at −2 V, (c) the outermost cathode ring at −70 V (V_out_, approximately equal to the full depletion voltage; Figure 8a) and −140 V (approximately two times the full depletion voltage, Figure 8b), and (d) the back side at −70 V (V_B_ = −70 V). The electric field inside the SDD is the vector sum of the longitudinal field (generated by the voltage difference between the back electrode VB and the front cathode ring) and the transverse field (generated by the linear voltage gradient along the front cathode ring chain).

As shown in Figure 8, the electric potential contours are well-defined and show clear paths toward the collection anode. The path marked by the arrow lines is even clearer, smoother, and well-defined. The range shown here is from −2500 μm to 100 μm, which results in a clearer visualization.

**Figure 9:**
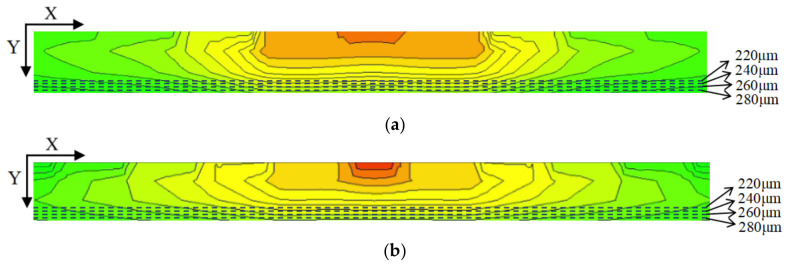
(**a**) Potential profile at one times the depletion voltage; (**b**) potential profile at two times the depletion voltage.

To observe the uniformity of the detector electric potential distribution in more detail, we further made cross-sectional cuts along a constant Y in Figure 9, specifically at Y = 280, 260, 240, and 220 µm.

**Figure 10:**
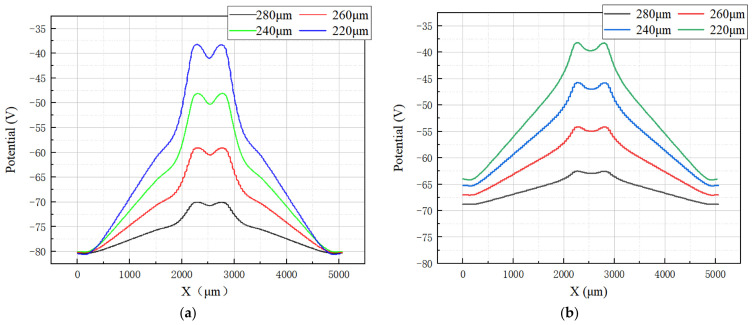
(**a**) One-dimensional potential distribution at V_out_ = one times the depletion voltage; (**b**) one-dimensional potential distribution at V_out_ = two times the depletion voltage.

Figure 10a,b show the one-dimensional electric potential distribution of the SDD along these cut lines at V_out_ equals one and at two times the depletion voltage, respectively. A relatively smooth potential distribution curve can be seen, and through these potential profiles, it is further confirmed that the path of charge collection is directed toward the center collection anode. Furthermore, calculations show that the average drift electric field is approximately 110 V/cm, representing an improvement of about 10% compared to conventional silicon drift detectors.

### 3.2. Detector Electric Field Distribution

**Figure 11:**
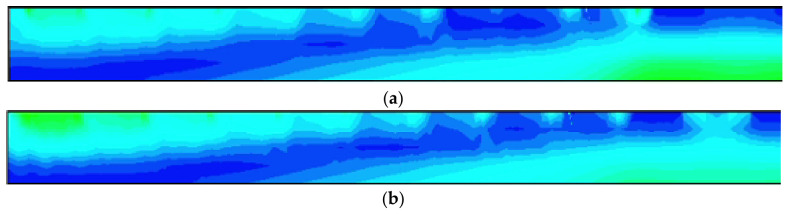
(**a**) Internal electric field distribution at one times the depletion voltage. (**b**) Internal electric field distribution at two times the depletion voltage.

Again using the cutting line shown in Figure 5, we can obtain the 2D electric field distribution map from the 3D-simulation data as shown in Figure 11. It is clear that the SDD distribution is fully depleted throughout the detector. To better illustrate the SDD’s electrical properties, we applied V_out_ = −70 V (approximately one times the depletion voltage) and −140 V (approximately two times the depletion voltage) to the outermost ring. It can be seen that there exist obviously low electric field (E-field) regions within the detector, which actually define the electron drift channels. The low E-field in the drift channels is mainly caused by the fact that it is dominated by the transverse E-field, at approximately ≥400 V/cm. In contrast, the E-field in regions outside the drift channels is higher than 600 V/cm, primarily dominated by the vertical E-field. Clearly, the electric field distribution of the SDD shown in Figure 10 is relatively smooth and well-defined.

**Figure 12:**
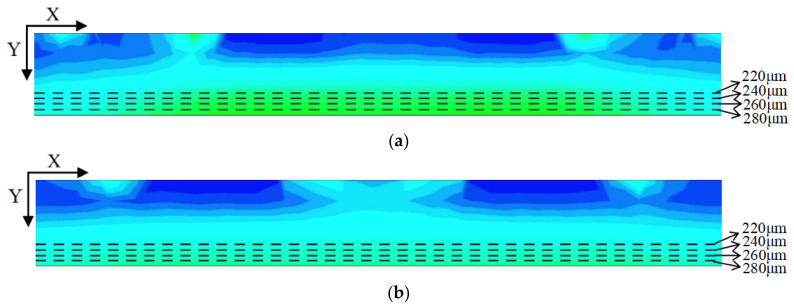
(**a**) Internal electric field distribution at one times the depletion voltage in center. (**b**) Internal electric field distribution at two times the depletion voltage in center.

In order to further quantitatively view the distribution of the detector electric field, we performed additional cuts at constant Y positions in Figure 12, specifically at Y = 280, 260, 240, and 220 µm.

**Figure 13:**
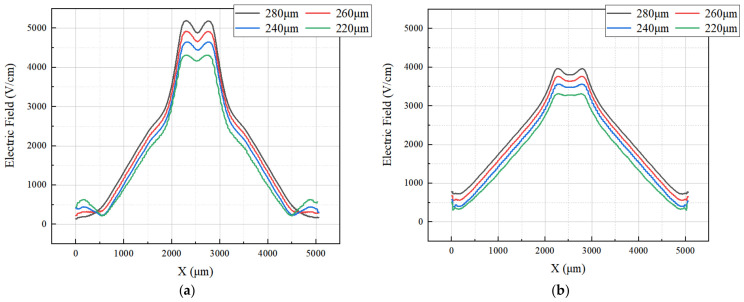
(**a**) One-dimensional electric field distribution at one times the depletion voltage; (**b**) one-dimensional electric field distribution at two times the depletion voltage.

Figure 13a,b show the one-dimensional electric field distribution of the SDD cross-section at V_out_ = one times and two times the depletion voltage, respectively. It is clearly observed that, both at V_out_ = −70 V and −140 V, these E-field curves are all greater than zero and relatively smooth, especially for V_out_ = −140 V case.

**Figure 14:**
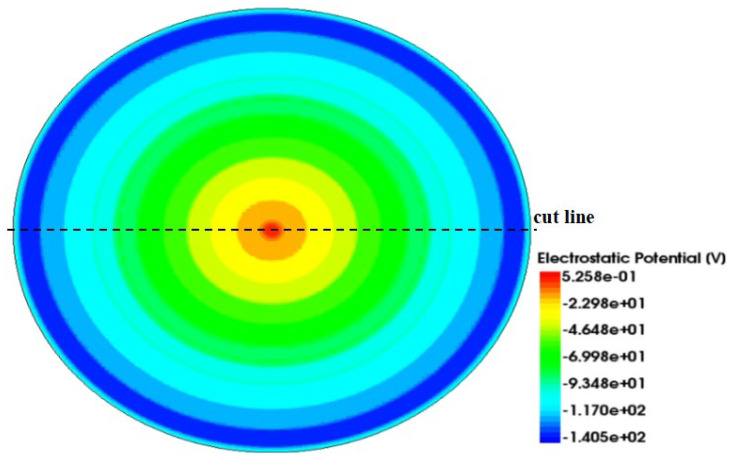
Cross-sectional potential diagram of the probe.

Figure 14 shows the cross-sectional potential of the detector at Z = 0.5 μm with a bias voltage of −140 V. The potential distribution is uniform, decreasing from the outer ring to the inner ring, indicating that the detector design meets the expected performance.

**Figure 15:**
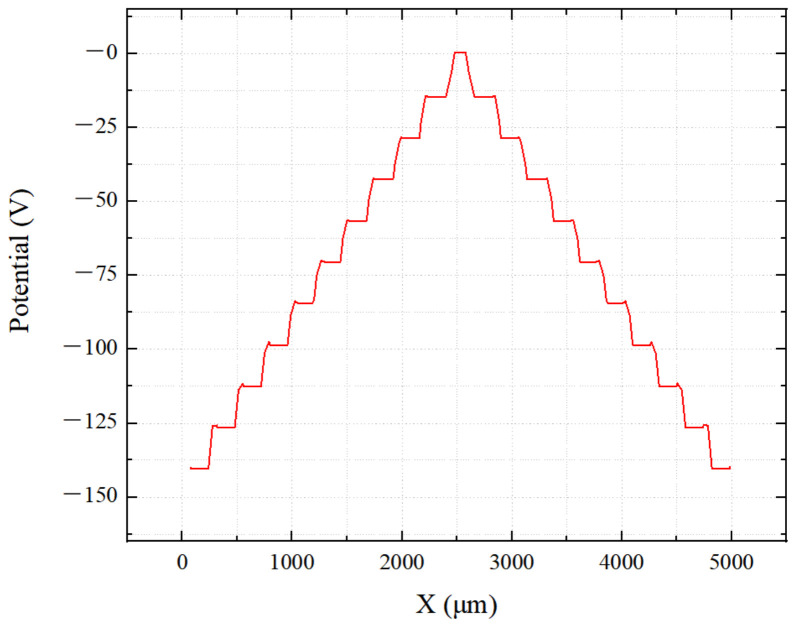
Surface potential distribution map of the probe.

Figure 15 is a one-dimensional image taken along the tangent shown in Figure 14, which more clearly shows that the potential gradient on the detector surface is more uniform and smooth, while the potential on the drift rings decreases stepwise, indicating that the SDD achieves a good automatic uniform voltage division, verifying the accuracy and rationality of the design calculations. Figure 15 shows a one-dimensional image taken along the tangential direction indicated in Figure 14. This figure more clearly demonstrates that the potential gradient on the detector surface is more uniform and smooth, while the potential on the drift ring decreases in a stepped pattern. This indicates that the SDD achieves effective automatic voltage division, thereby verifying the accuracy and validity of the design calculations. Furthermore, compared to traditional concentric ring SDDs, the inter-ring voltage drop in this study remains highly uniform at twice the depletion voltage, amounting to only approximately 10 V.

## 4. Conclusions

Based on the concentric circular SDD structure, this work proposes and develops a sectional-chain silicon drift detector with an elliptical-shaped voltage-dividing resistor chain. Using the Sentaurus TCAD semiconductor device simulation software, one 1/6 section of a whole circular detector was assembled into a complete circular SDD, and the electrical properties of the SDD were simulated (full 3D simulation). Simulation results indicate that this new SDD has a smooth surface potential distribution and a smooth and complete electric field distribution with a near constant lateral E-field in a well-defined electron drift channel. The electron concentration diagram reveals the gradual depletion of high-electron-concentration regions under optimal bias, forming a clear drift channel toward the anode. Future improvement of our SDD uniformity can be achieved by increasing the number of rings through adjusting the value of parameter m_N_ (the number of elliptical resistor arcs in the Nth stage of the elliptical resistor chain).

This work provides verification of the new SDD’s electrical performance. However, a more comprehensive verification of dynamic performance—including energy resolution—will rely on subsequent fabrication tests and more complex system-level simulations, which will be the focus of future work.

In future works, we will also study how the incidence position of high-energy particles affects signal collection—for example, by changing the distance between the incidence position and the collecting anode. We will also vary V_B_ and V_out_ to examine the effect of different bias voltages on the drift channel. Finally, we will determine the optimal structural configuration of the SDD. This new computational and design approach can provide valuable insight for the future design and fabrication of SDD devices.

## Data Availability

The original contributions presented in this study are included in the article. Further inquiries can be directed to the corresponding authors.
